# Acupuncture for anxiety: A protocol for a systematic review of controlled trials

**DOI:** 10.1097/MD.0000000000010266

**Published:** 2018-04-06

**Authors:** Sung-Youl Choi, Geun-Woo Kim

**Affiliations:** a Department of Neuropsychiatry, College of Korean Medicine, Gachon University; b Department of Neuropsychiatry, College of Korean Medicine, Dongguk University, Republic of Korea.

**Keywords:** acupuncture, anxiety, protocol, systematic review

## Abstract

**Background:**

Acupuncture has been widely, used in Asian countries since the Yuan Dynasty in China. Moreover, acupuncture has been reported to exhibit anti-allergy effects in many clinical trials. This systematic review will assess the effectiveness, and safety of acupuncture for anxiety treatment.

**Methods:**

Eleven databases, including Asian databases, will be searched for studies conducted through December in the year 2017. We will include randomized controlled trials (RCTs) assessing acupuncture for anxiety. The risk of bias will be evaluated using the Cochrane risk of bias assessment tool, and confidence in the cumulative evidence will be evaluated using the Grading of Recommendations Assessment, Development, and Evaluation (GRADE) instrument.

**Ethics and dissemination:**

This systematic review will be published in a peer-reviewed journal. It will also be disseminated electronically, and in print. The review will be updated to inform, and guide healthcare practices.

**Registration number:**

PROSPERO 2018 under number CRD42018080034.

## 1 Introduction

Anxiety disorder is a psychiatric disorder that is most commonly encountered accompanying depression in primary medical institutions.^[1]^ In particular, Koreans must adapt to various values, and rapid changes. A high prevalence of anxiety disorder is observed in the Korean population, which is directly, or indirectly, exposed to rapid, and chronic stress. Previous studies have shown that anxiety disorders lead to increased morbidity rates for other mental, and physical disorders, increased costs for health care services, and psychosocial impairment. Therefore, there is a continuing need to diagnose anxiety disorder early, and treat it without delay.^[2]^ In particular, the somatic symptoms of anxiety disorders are easily overpowered by other diseases, because of their great diversity. Psychological symptoms are not limited to specific episodes; rather, they are caused by interactions of various factors, such as individual personality characteristics, and the surrounding social environment, such that it is difficult to diagnose, and manage anxiety in the clinical field.

In modern medicine, somatic symptoms, and psychological symptoms of anxiety disorders are no longer independent, or individual.^[3]^ In the 10th revision of the International Statistical Classification of Diseases, and Related Health Problems (ICD-10), the tradition of clearly, distinguishing between neurosis, and psychosis has been abandoned, and theterm “neurotic” has been replaced by “neurotic, stress-related, and somatoform disorders.” Anxiety disorder is a representative disease of this “neurotic, stress-related, and somatic disorder” category, and is a disease that may strongly, benefit from the use of the oriental medicine theory with the viewpoint of “mind, and body,” which emphasizes the correlation between the mind, and the body.^[4]^

Patients with anxiety disorders have higher psychological attributes than do those with other conditions. Acupuncture is now commonly used in clinical practice, and is advantageous because it is less convenient, and less dependence-inducing (and thereby safer) than standard psychoactive drugs.^[5]^ Acupuncture treatment can be performed in a variety of manners, including general acupuncture, and the studies on these methods vary accordingly.^[6–8]^ In designing this protocol, we aimed to facilitate a review of acupuncture effectiveness for anxiety.

## 2 Methods

### 2.1 Study registration

This study will follow the guidelines outlined in the Preferred Reporting Items for Systematic Reviews, and Meta-Analysis (PRISMA) statement for meta-analyses of healthcare interventions^[9]^; additionally, the current protocol report adheres to the PRISMA Protocols (PRISMA-P) guidelines.^[10]^ The protocol for this systematic review has been registered in PROSPERO 2018 under number CRD42018080034.

### 2.2 Ethical consideration

This study is not a clinical study. Therefore, ethical approval is not needed.

### 2.3 Data sources

The following databases will be searched from inception to the present date: Medical Literature Analysis, and Retrieval System Online (MEDLINE), Excerpta Medica Database, the Cochrane Central Register of Controlled Trials (CENTRAL), Allied, and Complementary Medicine Database (AMED), and Cumulative Index to Nursing, and Allied Health Literature (CINAHL). We will also search 6 Korean medical databases (Online Archiving & Searching Internet Sources [OASIS], the Korean Traditional Knowledge Portal, the Korean Studies Information Service System, KoreaMed, the Korean Medical Database, and DBpia), and 3 Chinese databases (China National Knowledge Infrastructure [CNKI], including the China Academic Journal, the China Doctoral Dissertations, and Master's Theses Full-text Database, the China Proceedings of Conference Full-Text Database, and the Century Journal Project; W and VIP). In addition, we will search a Japanese database, and conduct non-electronic searches of conference proceedings, our own article files, and 9 traditional Korean medical journals.

## 3 Types of studies

Prospective randomized controlled trials (RCTs) that evaluate the effectiveness of acupuncture in reducing anxiety will be included in this review. Both treatment with acupuncture alone, and concurrent treatment with acupuncture, and another therapy will be considered acceptable if acupuncture is applied to the intervention group only, and any other treatment is provided equally to both the groups. Trials with any type of control intervention will be included. There will be no restrictions on publication language.

## 4 Types of participants

Diverse groups of patients with anxiety will be eligible for inclusion. Participants who have both anxiety, and co-morbid diseases will be excluded. There will be no restrictions based on other conditions, such as age, sex, or symptom severity.

## 5 Types of interventions

Studies that evaluate any type of invasive acupuncture, with, or without electrical stimulation, will be included. The treatments used in the studies must involve needle insertion at acupuncture points, pain points, or trigger points, and must be described as Acupuncture. Control interventions may include general conventional care (including drugs, and behavioral treatments), sham treatments (interventions mimicking “true” acupuncture/ “true” treatment, but deviating in atleast 1 aspect considered important by Acupuncture theory, such as skin penetration, or correct point location), or waiting list care.

## 6 Data extraction and quality assessment

Hard copies of all articles will be obtained, and read in full. Two authors (SC and GWK) will perform the data extraction, and quality assessment using a predefined data extraction form. In addition, detailed methods of acupuncture will be extracted, using the Standards for Reporting Interventions in Clinical Trials of Acupuncture (STRICTA) guidelines. The risk of bias will be evaluated using the Cochrane risk of bias assessment tool, version 5.1.0, which considers random sequence generation, allocation concealment, blinding of participants, and personnel, blinding of outcome assessment, completeness of outcome data, selective reporting, and other sources of bias.^[11]^ The results of such evaluations will be presented by utilizing scores of “L,” “U,” and “H” to indicate a low risk of bias, an uncertain risk of bias, and a high risk of bias, respectively. Disagreements will be resolved by discussion amongst all authors. When disagreements regarding selection cannot be resolved through discussion, an arbiter will make the final decision.

## 7 Data collection and synthesis

### 7.1 Outcome measures

#### 7.1.1 Primary outcomes

The primary outcomes will be the therapeutic effects of treatment on anxiety.

#### 7.1.2 Secondary outcomes

The secondary outcomes will include safety, which will be evaluated based on adverse effects.

### 7.2 Assessment of bias in the included studies

We will independently assess bias in the included studies in accordance with criteria in the Cochrane Handbook, version 5.1.0; these criteria include random sequence generation, allocation concealment, blinding of participants, and personnel, blinding of outcome assessment, completeness of outcome data, selective reporting, and other sources of bias.^[11]^

### 7.3 Data synthesis

Differences between the intervention, and control groups will be assessed. Mean differences (MDs) with 95% confidence intervals (CIs) will be used to measure the effects of treatment for continuous data. We will convert other forms of data into MDs. For outcome variables on different scales, we will use standard MDs (SMDs) with 95% CIs. For dichotomous data, we will present treatment effects as relative risks (RRs) with 95% CIs; other binary data will be converted into RRs.

All statistical analyses will be conducted using the Cochrane Collaboration's software program, Review Manager (RevMan) for Windows (Version 5.3. Copenhagen: The Nordic Cochrane Centre, The Cochrane Collaboration, 2014). We will contact the corresponding authors of studies with missing information to acquire, and verify data whenever possible. As appropriate, we will pool data across studies to conduct a meta-analysis using fixed-, or random-effects modeling. We will use the GRADEpro software from Cochrane Systematic Reviews to create a “summary of findings” table.

### 7.4 Unit of analysis issues

For crossover trials, data from the 1^st^ treatment period will be used. For trials that assessed more than 1 control group, the primary analysis will combine data from all control groups. Subgroup analyses of the control groups will be performed. Each patient will be counted only once in these analyses.

### 7.5 Addressing missing data

Intention-to-treat analyses that include all randomized patients will be performed. For patients with missing outcome data, last observation carry-forward analysis will be conducted. When individual patient data are initially unavailable, we will review the original source, and/or published trial reports to obtain these data.

### 7.6 Assessment of heterogeneity

Following our initial data analyses, we will use random-, or fixed-effects models to conduct the meta-analysis. Chi-squared, and I-squared tests will be used to evaluate the heterogeneity of the included studies, with an I^2^ of >50% considered indicative of high heterogeneity. When heterogeneity is observed, subgroup analyses will be conducted to explore the possible causes of this heterogeneity.^[12]^

### 7.7 Assessment of reporting biases

Funnel plots will be generated to detect reporting biases when a sufficient number of included studies (at least 10 trials) are available.^[13]^ However, because funnel plot asymmetries are not equivalent to publication biases, we will aim to determine possible reasons for any asymmetries in the included studies; potential causes of such asymmetries include small-study effects, poor methodological quality, and true heterogeneity.^[13,14]^

## 8 Discussion

Acupuncture is widely used in various Asian countries. This systematic review will provide evidence for the use of acupuncture in the treatment of anxiety.

## Author contributions

SC, and GWK conceived the study, developed the study criteria, searched the literature, analyzed the data, and wrote the protocol. GWK conducted the preliminary search. SC assisted in searching the Chinese literature, and extracting data. GWK revised the manuscript. All authors have read, and approved the final manuscript.

**Conceptualization:** S.Y. Choi.

**Funding acquisition:** G-W. Kim.

**Investigation:** G-W. Kim.

**Methodology:** G-W. Kim.

**Writing – original draft:** S.Y. Choi.

**Writing – review & editing:** G-W. Kim.
